# 431. SARS-CoV-2 Environmental Contamination in Hospitalized COVID-19 Patients’ Rooms

**DOI:** 10.1093/ofid/ofab466.631

**Published:** 2021-12-04

**Authors:** Bobby G Warren, Alicia Nelson, Aaron Barrett, Bechtler S Addison, Amanda M Graves, Sarah S Lewis, Becky A Smith, David J Weber, David J Weber, Emily Sickbert-Bennett, Deverick J Anderson

**Affiliations:** 1 Duke Center for Antimicrobial Stewardship and Infection Prevention, Durham, North Carolina; 2 Duke University, Durham, NC; 3 Duke School of Medicine, Cary, North Carolina; 4 University of North Carolina, Chapel Hill, NC; 5 UNC Health Care, Chapel Hill, NC

## Abstract

**Background:**

The correlation between SARS-CoV-2 RNA and infectious viral contamination of the hospital environment is poorly understood.

**Methods:**

housed in a dedicated COVID-19 unit at an academic medical center. Environmental samples were taken within 24 hours of the first positive SARS-CoV-2 test (day 1) and again on days 3, 6, 10 and 14. Patients were excluded if samples were not obtained on days 1 and 3. Surface samples were obtained with flocked swabs pre-moistened with viral transport media from seven locations inside (bedrail, sink, medical prep area, room computer, exit door handle) and outside the room (nursing station computer). RNA extractions and RT-PCR were completed on all samples. RT-PCR positive samples were used to inoculate Vero E6 cells for 7 days and monitored for cytopathic effect (CPE). If CPE was observed, RT-PCR was used to confirm the presence of SARS-CoV-2.

**Results:**

We enrolled 14 patients (Table 1, Patient Characteristics) between October 2020 and May 2021. A total of 243 individual samples were obtained – 97 on day 1, 98 on day 3, 34 on day 6, and 14 on day 10. Overall, 18 (7.4%) samples were positive via RT-PCR – 9 from bedrails (12.9%), 4 from sinks (11.4%), 4 from room computers (11.4%) and 1 from the exit door handle (2.9%). Notably, all medical prep and nursing station computer samples were negative (Figure 1). Of the 18 positive samples, 5 were from day 1, 10 on day 3, 1 on day 6 and 2 on day 10. Only one sample, obtained from the bedrails of a symptomatic patient with diarrhea and a fever on day 3, was culture-positive (Figure 2).

Table 1. Patient Characteristics

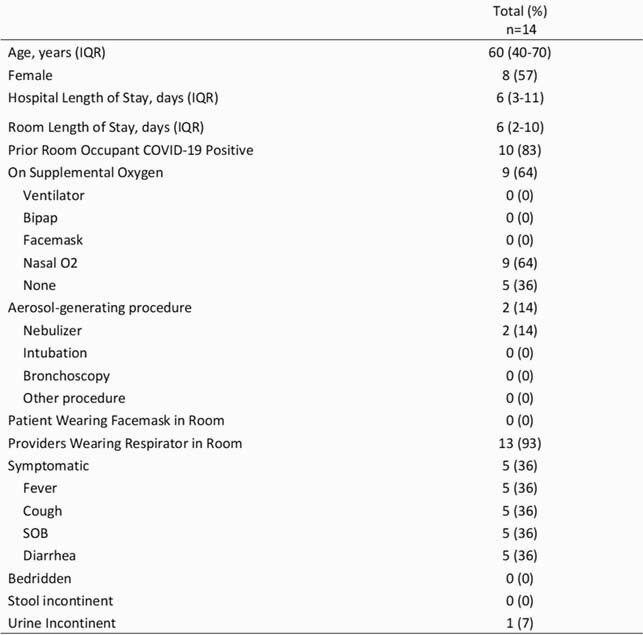

Figure 1. Proportion of RT-PCR Positive Samples by Sample Day and Location

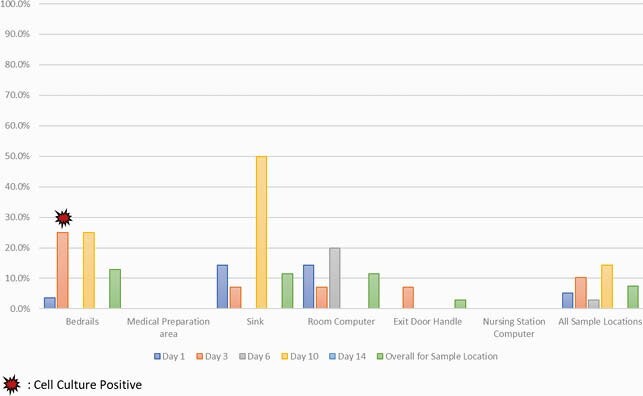

Figure 2. Cell cultures of negative control (left) and CPE positive sample (right)

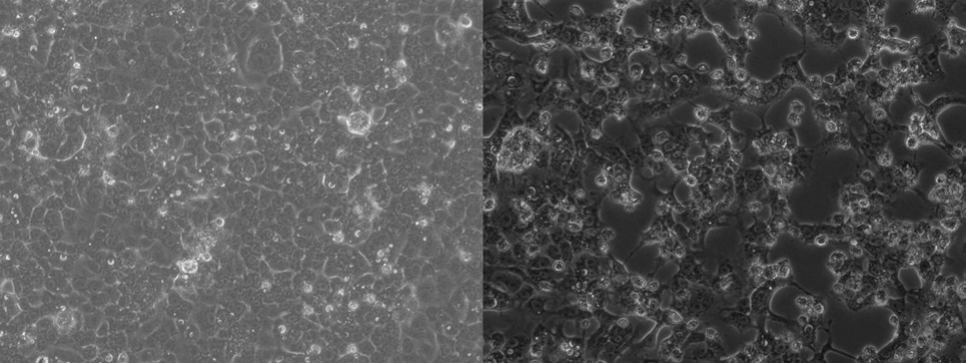

**Conclusion:**

Overall, the amount of environmental contamination of viable SARS-CoV-2 virus in rooms housing COVID-19 infected patients was low. As expected, more samples were considered contaminated via RT-PCR compared to cell culture, supporting the conclusion that the discovery of genetic material in the environment is not an indicator of contamination with live infectious virus. More studies including RT-PCR and viral cell culture assays are needed to determine the significance of discovering SARS-CoV-2 RNA versus infectious virus in the clinical environment.

**Disclosures:**

**David J. Weber, MD, MPH**, **PDI** (Consultant)

